# How Current Are Leading Evidence-Based Medical Textbooks? An Analytic Survey of Four Online Textbooks

**DOI:** 10.2196/jmir.2105

**Published:** 2012-12-10

**Authors:** Rebecca Jeffery, Tamara Navarro, Cynthia Lokker, R Brian Haynes, Nancy L Wilczynski, George Farjou

**Affiliations:** ^1^Health Information Research UnitDepartment of Clinical Epidemiology and BiostatisticsMcMaster UniversityHamilton, ONCanada

**Keywords:** databases, bibliographic, medical informatics, evidence-based medicine

## Abstract

**Background:**

The consistency of treatment recommendations of evidence-based medical textbooks with more recently published evidence has not been investigated to date. Inconsistencies could affect the quality of medical care.

**Objective:**

To determine the frequency with which topics in leading online evidence-based medical textbooks report treatment recommendations consistent with more recently published research evidence.

**Methods:**

Summarized treatment recommendations in 200 clinical topics (ie, disease states) covered in four evidence-based textbooks–UpToDate, Physicians’ Information Education Resource (PIER), DynaMed, and Best Practice–were compared with articles identified in an evidence rating service (McMaster Premium Literature Service, PLUS) since the date of the most recent topic updates in each textbook. Textbook treatment recommendations were compared with article results to determine if the articles provided different, new conclusions. From these findings, the proportion of topics which potentially require updating in each textbook was calculated.

**Results:**

478 clinical topics were assessed for inclusion to find 200 topics that were addressed by all four textbooks. The proportion of topics for which there was 1 or more recently published articles found in PLUS with evidence that differed from the textbooks’ treatment recommendations was 23% (95% CI 17-29%) for DynaMed, 52% (95% CI 45-59%) for UpToDate, 55% (95% CI 48-61%) for PIER, and 60% (95% CI 53-66%) for Best Practice (*χ* ^*2*^_*3*_=65.3, *P*<.001). The time since the last update for each textbook averaged from 170 days (range 131-209) for DynaMed, to 488 days (range 423-554) for PIER (*P*<.001 across all textbooks).

**Conclusions:**

In online evidence-based textbooks, the proportion of topics with potentially outdated treatment recommendations varies substantially.

## Introduction

Online evidence-based textbooks such as UpToDate, Physicians’ Information Education Resource (PIER), DynaMed, and Best Practice aim to provide high quality, frequently updated, evidence-based recommendations for clinical practice, enabling clinicians to provide care to their patients, meeting the most recent evidence-based standards [1, 2]. If these goals are achieved, they offer many advantages for supporting clinical decisions, especially in indicating care based on the current best evidence [3]. However, these textbooks often contain topic summaries that have not been recently updated [4-7]. New evidence potentially important to advancing medical practice is published frequently at unpredictable rates, making it necessary for frequent reassessment of management recommendations [5,6]. Lag time between publication of research and its availability in textbooks puts a burden on clinicians, who may provide suboptimal care to patients as a consequence [3].

It is very costly and time consuming for evidence-based textbooks to seek out, appraise, and incorporate new information in a timely manner, using only their own resources [8]. Improved efficiency at lower costs would be an asset to updating such textbooks, and perhaps free-up resources to tackle challenges in integrating evidence-based information into clinical work flow. Tools such as specific and sensitive literature search filters and evidence-based literature appraisal services could help overcome these barriers [9]. Recent studies have assessed the time since updating of individual topics within evidence-based textbooks [4,7,10] but have not attempted to identify individual studies that might affect recommendations for care and thus warrant considering a revision of the most recent textbook version.

The McMaster Premium Literature Service (PLUS) database is a continuously updated, searchable database of primary studies and systematic reviews. Each article from over 120 high quality clinical journals and evidence summary services, such as the AHRQ Technology Assessment Program, is appraised by research staff for methodological quality, and articles that pass basic criteria are assessed by practicing clinicians in the corresponding discipline [1, 9]. Clinical ratings are based on 7-point scales, where clinical relevance ranges from 1 (“not relevant”) to 7 (“directly and highly relevant”), and newsworthiness ranges from 1 (“not of direct clinical interest”) to 7 (“useful information, most practitioners in my discipline definitely don’t know this”). Recently, use of the PLUS database on its own has been shown to identify articles that can be used to update a high proportion of systematic reviews [11].

The primary objective of this investigation was to determine the proportion of topics in UpToDate, PIER, DynaMed, and Best Practice that predated articles in PLUS with findings different from those reported in the topics. We also assessed the number of topics available in each evidence-based textbook compared with the topic coverage in the PLUS database and the recency of updates for these publications.

## Methods

### Study Design

An analytic survey of 200 clinical topics across four online evidence-based textbooks was performed based on their most recent update to determine how frequent these topics omitted evidence in PLUS that was published since the most recent textbook update. The four textbooks, UpToDate, PIER, DynaMed, and Best Practice, were selected based on their performance in a study which assessed their evidence-based standards of preparation, timeliness of updating, and breadth of topic coverage [4]. These four textbooks have online referencing for treatment recommendations and were available through the McMaster PLUS Federated Search website [12], allowing for simultaneous searching of all textbooks for each topic.

### Topic Selection

Articles in the PLUS database are indexed for over 700 topics, 502 of which had at least one corresponding article in the database in June 2011. A randomized sequence for the 502 topics was generated for topic selection. To permit comparisons of the textbooks, topics from the randomized sequence were included if each of the four textbooks had a corresponding chapter or section, and each included treatment recommendations. Searches were conducted using the textbooks’ search engine via a federated search platform [12] that searched the four textbooks simultaneously, using search terms derived from Systematized Nomenclature of Medicine (SNOMed) coding of the topic names for the 200 topics. Topics were excluded if there were more than 5 links to related chapters within a textbook, because it would be difficult to analyze all the relevant pages to this many external links. Topics were assessed for eligibility until our sample size of 200 topics was reached.

### Status of Updating for Topics

Corresponding chapters for each topic in each textbook were captured as PDFs at the identical time for a given topic to provide a common baseline capture date for all texts, from July 2011 to November 2011. Any additional chapters linked to specific treatments for the topics of interest (up to 5 links in total) were also captured. It was not feasible to conceal the textbook sources of the PDFs from research staff (RJ, GF, TN) doing assessments; however, the protocol was standardized and outcomes were objective to minimize bias introduced by this lack of blinding. 

The date of the most recent article cited for a topic was used as the update time since the textbooks did not consistently specify whether the posted date referred to the last literature search, the date the update was posted, or otherwise.

A simultaneous search of the PLUS database was conducted, using the pre-assigned SNOMed indexing terms for each topic, and including only articles that reported randomized trials or systematic reviews of trials relevant to questions about therapy. Data captured included PubMed ID, article title and abstract, and the date the article was posted to PLUS. The PLUS posting date was compared to the last update for each textbook to determine which article was more recent.

The treatments studied in the selected articles were compared to those recommended in the textbooks. If the study findings were qualitatively different from the textbook recommendation, the findings in the article were considered as potential update material for a textbook. For example, if a PLUS article reported a significant effect of nebulized epinephrine for croup and a textbook did not recommend this therapy, the article would be deemed a potential update for this topic. The term potential update was used to acknowledge the fact that clinical recommendations require the consideration of accumulated evidence, patients’ circumstances, and other variables such as the quality of the new evidence, in addition to the new evidence itself.

Articles were assessed to determine if the findings of the study showed a benefit, harm, or no effect of the treatment on the clinical outcome reported in the study. This assessment was initially done in duplicate by 2 reviewers (RJ and TN) for 20 topics. The rate of agreement beyond chance (kappa statistic) was 91% (81%-99%). Subsequently, only 1 reviewer assessed each article for the other 180 topics.

The proportion of topics in each textbook with the potential to update was the primary outcome of interest. A topic was in need of an update if there was at least one newer article in PLUS that provided information that differed from the topic’s recommendations in the textbook. Practical importance was defined as textbooks which require an update on 15% or more of the topics. The sample size of 200 topics was determined based on this parameter with a 95% confidence interval of ±5%. Chi-squared tests were performed to determine if the proportions of topics needing updates for each textbook were significantly different across the four textbooks. Analysis of variance was used for continuous data comparisons. Stata version 9.2 (StataCorp LP, College Station, TX) was used for statistical analysis.

## Results

### Topic Selection

**Figure 1:**
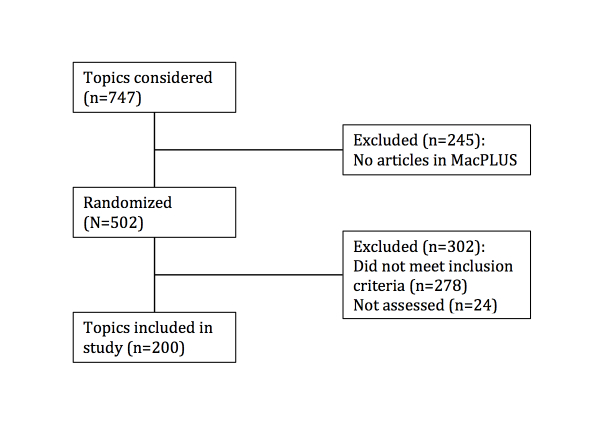
Flow Diagram of Study Population.

During topic selection, 478 topics of the 502 were assessed for eligibility. 271 topics were excluded because they were not found in all evidence based textbooks while 7 were excluded because they had more than 5 topic links in a single textbook (Figure 1). Of the excluded topics, a matching chapter was not available in PIER for 87% (241/278), Best Practice for 34% (95/278), DynaMed for 23% (63/278), and UpToDate for 19% (52/278), (*χ* ^*2*^_*3*_=345, *P*<.001 for the differences across textbooks).

### Status of Updating for Topics

Overall, 956 articles found in PLUS were more recent than the last update in at least one textbook over all 200 topics. 165 topics of the 200 had at least 1 article for at least 1 textbook that could be a potential update to that topic. All textbooks had >15% of topics with the potential for an update, ranging from 23% (46/200) for DynaMed to 60% (200) for Best Practice (*χ* ^*2*^_*3*_=65.3, *P*<.001, Table 1). The proportion of topics with potential for updates was significantly lower for DynaMed than the other three textbooks, which had statistically similar values (Table 1).

The trend was the same for the mean number of articles available in PLUS since the last textbook update; there were fewer articles accumulating for DynaMed compared with the other textbooks (Table 1). This is partly explained by the time since the last update. For DynaMed topics, updates occurred on average of 170 days prior to our study, while the other textbooks averaged from 427 to 488 days (Table 1).

Of all evidence-based textbooks, DynaMed missed fewer articles reporting benefit or no effect when the direction of findings available to update (beneficial, harmful, no effect) was investigated (Table 2). For articles reporting harm, there were fewer PLUS articles accruing for DynaMed than Best Practice; no other textbooks showed differences (Table 2).

The clinical relevance and newsworthiness for each article available in PLUS was also investigated, based on the score out of 7 for each category assigned to that article by PLUS raters. For the 406 accruing articles that reported new information on benefit of a treatment for at least 1 textbook, mean clinical relevance scores were 5.51 (95% CI 5.45-5.57; 7-point scale, with 7 high) and mean newsworthiness was 4.98 (4.91-5.04, 7-point scale). Mean clinical relevance scores were ≥ 6 out of 7 for 25% (101/406) of the studies; mean newsworthiness scores were ≥ 6 out of 7 for 9.4% (38/406) of these beneficial studies. For the 27 articles reporting harm, mean clinical relevance was 5.49 (5.24-5.74) and newsworthiness was 5.01 (4.72-5.31). Of the 27 articles reporting harm, 15% (4/27) were rated ≥ 6 for newsworthiness, 26% (7/27) were rated ≥ 6 for clinical relevance.

**Table 1:** Results for 200 equivalent topics across four evidence-based textbooks.

Variable	Best Practice	DynaMed	PIER	UpToDate
Number of topics with potential for updates	119	46	109	104
Proportion of 200 topics with potential for updates (95% CI)^a^	60% (53%-66%)^b^	23% (17%-29%)	55% (48%-61%) ^b^	52% (45%-59%) ^b^
Mean (CI) number of articles per topic in PLUS since last update^c^	1.94 (1.46-2.42) ^b^	0.48 (0.29-0.67)	1.56 (1.21-1.91) ^b^	1.67 (1.25-2.09) ^b^
Mean (CI) number of days since last update^c^	435 (392-478) ^b^	170 (131-209)	488 (423-554) ^b^	427 (360-494) ^b^

^a^ *χ* ^*2*^_*3*_=65.3, *P*<.001 comparing the four textbooks.

^b^Values sharing a superscript ^b^ are not significantly different from one another at the *P*=.01 (Bonferroni correction).

^c^One-way ANOVA *P*<.001.

**Table 2:** Subgroup analysis of articles in PLUS reporting harm, benefit, or no effect.

Variable	Best Practice	DynaMed	PIER	UpToDate
Mean (95% CI) number of new articles reporting benefit per 100 topics^a^	125 (88-161) ^b^	31 (19-43)	100 (75-124) ^b^	107 (075-138) ^b^
Mean (CI) number of new articles reporting harm per 100 topics^a^	14 (8-20)^b^	3 (0-5) ^c^	8 (4-12) ^bc^	11 (5-16) ^bc^
Mean (CI) number of new articles reporting no effect per 100 topics^a^	58 (42-73) ^b^	15 (6-24)	50 (35-64) ^b^	53 (38-68) ^b^

^a^One-way ANOVA *P*=.007 for number of new articles reporting harm and *P*<.001 for other outcomes comparing the textbooks.

^b,c^Values sharing a superscript^ b ^or^ c ^ are not significantly different from one another at *P*=.01 (Bonferroni correction).

## Discussion

We found that topic coverage varied substantially for leading evidence-informed electronic textbooks and generally a high proportion of the 200 common topics had potentially out of date conclusions, missing information from 1 or more recently published studies. PIER had the least topic coverage, while UpToDate, DynaMed, and Best Practice covered more topics, in similar numbers. DynaMed’s timeline for updating was the quickest and it had by far the least number of articles that needed to be updated, indicating that quality was not sacrificed for speed.

These findings were similar to a recent study looking at updating systematic reviews [11]. Our sample size included a large number of topics and provided power for our estimate of topics requiring potential updates. Indeed, we found a much higher rate of topics with a potential update than our pre-study expectation of 15%. Although the primary objective was to estimate updating potential within textbooks, some differences across textbooks were apparent.

Evidence-based textbooks are an important source of summary information and care recommendations for practicing clinicians [9]. Keeping these resources updated is a costly and intensive process. The results of this study show that although there is variation in the rate at which the leading textbooks are updated, all of them can benefit from more frequent processing of high quality, clinically relevant, recently published studies. The PLUS database, compared to the latest updates of 200 topics across textbooks, had studies with new, different information related to over half the topics for textbooks, and 23% for DynaMed topics. Some of these more recent articles contained information on benefits of treatment, as well as the potential for harm. This is an underestimate of the potential for PLUS to update textbooks across clinically important topics, as we did not consider studies of diagnosis, prognosis, clinical prediction, quality improvement, or cost-effectiveness, all of which are included in the PLUS database and may affect clinical decisions.

The articles identified as potential updates reporting benefits and harms had a range of scores for clinical relevance and newsworthiness, with most articles rated as “probably” to “definitely” relevant to clinical practice across disciplines, and rated as “useful information” by practicing physicians [13].

Studies have shown that patients often do not receive the best care, or may even receive harmful or unnecessary care, due to difficulties in updating information for practice [10]. Recently published articles about ineffective or potentially harmful treatments should also be included in recommendations, as physicians may not realize there are recent studies that contradict previous evidence. For example, in our study, percutaneous angioplasty for renal artery stenosis was found to be harmful in the ASTRAL trial [14], though the evidence was previously unclear. This trial, published in November 2009, was not cited in PIER, which had been updated for this topic in December 2009 at the time of our study; it has since been incorporated into PIER. At the time of our study, DynaMed, Best Practice, and UpToDate had updated renal artery stenosis to include the ASTRAL trial [14] and recommended against this procedure. Another example, recombinant activated factor VII was found to be harmful in spontaneous intracerebral hemorrhage, but had not yet been included in Best Practice, which was last updated on January 11, 2009, at the time of our study [15]. Therefore, these 2 examples were considered as potential updates from PLUS for textbooks.

It is important to note that no single study is likely to change a clinical recommendation on its own [16,17]. However, articles that we have indicated to be potential updates may tip the balance leading to a recommendation change. That said, creating evidence-based recommendations is a complex process requiring clinical expertise and judgment, and consideration of benefits, harms, costs, and patient values and preferences. For this reason, we are only able to report how many articles might potentially provide updates and cannot be certain that a high quality article from PLUS would impact textbook recommendations and necessitate an update.

The study appraised only online textbooks, each with varying chapter headings and coverage. Comparing the textbooks for currency required finding topics common to all four textbooks, which was challenging. In most instances when a topic was not included in our sample, it was because PIER did not have a comparable chapter or section, making it unlike the other textbooks in terms of breadth of content. Importantly, topics were excluded if they were not included in all textbooks, thus all topics in our study were covered in PIER.

Evidence-based clinical textbooks are in evolution, and we assessed them at only one timepoint, however we compared the updatedness of a large number of topics, allowing us to establish a clear picture of the updating practices of each textbook. We hope that they will continue to improve their coverage and timeliness in considering newly published evidence. All textbooks have access to the PLUS database to facilitate updates, and also use other sources of updates, such as practice guidelines. In this investigation, we looked only at studies published following the most recent textbook updates, and could not discern if the PLUS service is supplementary to other sources of appraised study reports. Redundancy abounds in evidence sources and resources, increasing the costs for publishers, editors, and authors to appraise, organize, and incorporate evidence in a timely fashion, unless ways of reducing redundancy are operationalized.

Future research should investigate best methods of facilitating efficient updates of medical textbooks and uptake of these practice changes by health care professionals. Our study documents that these textbooks have some ways to go in keeping pace with high quality, clinically relevant new evidence. This new evidence has the capacity to impact their clinical recommendations, and potentially the quality of patient care. Follow-up studies to document the progress of these texts in keeping pace with new evidence would be informative.

## Abbreviations

PLUS: McMaster Premium Literature Service
SNOMed: Systematized Nomenclature of Medicine
PIER: Physicians’ Information Education Resource
